# Editorial: Navigating complexity in postgraduate/graduate health professions education: innovative pedagogical approaches and assessment strategies

**DOI:** 10.3389/fmed.2025.1734397

**Published:** 2025-11-25

**Authors:** Shaista S. Guraya, Preman Rajalingam, Nabil Zary

**Affiliations:** Institute of Learning (IoL), Mohammed Bin Rashid University of Medicine and Health Sciences (MBRU), Dubai Health, Dubai, United Arab Emirates

**Keywords:** health professions education, postgraduate medical education, pedagogy, assessment, graduate medical education

Some perceive postgraduate education as the ‘stepchild' of health professions education (HPE), receiving less systematic attention than its undergraduate counterpart. While institutions often remain preoccupied with the minute details of undergraduate curricula, they often remain oblivious to the rapidly shifting landscape, expectations, and complexities of the postgraduate realm. Yet, this is the very stage where professional identities are refined, clinical complexity is lived, and educational interventions must move beyond replication of undergraduate strategies. Our special Research Topic “*Navigating complexity in postgraduate/graduate health professions education: innovative pedagogical approaches and assessment strategies”* brings together eleven pieces that examine this neglected space, focusing on curricular and pedagogical innovations, particularly in contexts where resource constraints, entrenched traditions, or disciplinary gaps have left postgraduate learning under-supported. Study designs range from curriculum reviews and qualitative inquiries to cross-sectional surveys and systematic reviews, mirroring the diversity of Research Topics in postgraduate learning. Together, they expose the cracks, confront old habits, and offer glimpses of what is possible when postgraduate education is treated not as an afterthought, but as a dynamic, intellectually demanding, and deeply human enterprise.

To guide readers through this diverse set of contributions, we have provided structured summaries with analytical commentary, highlighting key insights and provocations emerging from each article.

## Reclaiming research education

In postgraduate medical education (PGME), research is often treated as a box to be ticked rather than a competency to be nurtured. Mwangi et al. offer a refreshing departure from this trend by presenting a longitudinal, structured research curriculum evaluation study for residents in a low-resource setting. The strength of this paper lies in its conceptual clarity. It addresses the fundamental ‘what, how, for whom, when, and how long' questions that many institutions gloss over. This model offers a framework that can be adapted across contexts, with careful attention to local needs, available expertise, and institutional capacity.

## Designing a pain curriculum

Mathew et al. identify pain as a threshold concept yet fragmented and under-theorized, anchoring it within a holistic, phased framework that deliberately integrates socio-cultural and psychological perspectives. The paper offers both a pedagogical blueprint and a provocation to witness how pain education might be globally reimagined. Using an action research approach, the authors created 22 h of e-learning materials aligned with international standards and freely disseminated to 469 physiotherapy institutions. The question now is whether such a model can be transplanted across borders with fidelity, a witnessing that could shape international curricula.

## To tutor, the tutor must be tutored

Clinician tutors occupy a unique and often precarious space in postgraduate education. Berlanga-Fernández et al. bring their voices to the fore through a qualitative descriptive study that explores the experiences and training needs of family medicine and nursing tutors surfacing a profound duality of identity. On one hand, tutors shoulder the heavy expectations of supporting learners; while themselves yearning for structured pedagogical guidance, emotional literacy, and institutional support. The authors' conclusion is both simple and profound, where we infer that ‘to tutor, the tutor must be tutored' reminding us that faculty development is not ancillary.

## The grand round as educational theater

The grand round has long been revered as the jewel of the CLE a ritual performed for centuries. Yet its emotional, cognitive, and performative complexities are rarely interrogated. Li et al. apply a qualitative design-based research method and shift the spotlight onto all three central actors: patients, clinicians, and learners. Within this deeply staged encounter, their qualitative exploration reveals strikingly parallel anxieties. Patients feel overwhelmed by medical jargon, clinicians fear exposing their technical or teaching vulnerabilities, and learners walk on eggshells, grappling with cognitive overload while striving to absorb clinical pearls and display empathetic presence. The study illuminates how grand rounds function as a high-stakes performance where psychological comfort, cognitive bandwidth, and emotional engagement collide. Confucian or not, the CLE remains a formidable beast.

## Knowledge curation and not just consumption

In specialties where knowledge evolves at breakneck speed, learners often spend more time curating resources than engaging with them. Koleva et al. use a mixed-methods comparative evaluation to examine the impact of a curated address this problem by introducing a comprehensive e-repository for physiatry education, designed as a centralized ‘one-stop shop' for graduate and postgraduate learners. Over four and a half years, nearly 2,000 learners engaged with this repository, which offered curated, discipline-specific content across neurorehabilitation, orthopedics, and other domains. The results were impressive. Significant gains in theoretical knowledge and practical performance, alongside exceptionally high learner satisfaction. What stands out is the elegant simplicity of the intervention, thoughtful curation, and open access rather than technological novelty.

## Four generations and one workplace

We are witnessing a truly unprecedented moment in healthcare and academia. Four generations; Baby Boomers, Generation X, Millennials, and Generation Z work together in shared spaces. Tumala et al. explore this reality through a cross-sectional survey of nursing faculty and staff in Saudi universities, examining attitudes and perceptions toward intergenerational workplace climates. Their findings reveal a moderately positive climate overall, with the strongest scores in affect and inclusiveness, but weaker intergenerational contact. While generational differences were not statistically significant, meaningful variations emerged across roles, education levels, and institutional contexts. CLEs are already complex, and layering intergenerational dynamics onto this complexity creates new tensions and opportunities.

## The stepchild exposed

Postgraduate education continues to struggle with inherited structures that no longer fit contemporary needs. Xu et al. bring this into sharp focus through their study of differences between academic and professional postgraduate programs in China, the world's second most populous country. Their survey of students and supervisors exposes misalignments in curriculum priorities, teaching modes, and expectations. Students lean toward research-based and online learning formats, while supervisors value blended professional coursework. Redundancy with undergraduate content was a repeated complaint, highlighting the structural and conceptual lag of postgraduate curricula.

## Gagne's promise but the design gap

Li et al. perform a systematic review and meta-analysis comparing nascently used Gagne's nine events of instruction with traditional lecture-based learning in HPE. The results are, on the surface, impressive, revealing significant gains in knowledge, practice, learning compliance, and teaching satisfaction. Yet, beneath these numbers lies a fuzzier picture. The trials themselves were small, heterogeneous, and vague about what exactly worked and why. This is where the paper is both revealing and provocative. We know structured approaches work better, but we don't know ‘how'. Learner-centered approaches like Gagne's hinge on stimulation, motivation, and active engagement. Future studies need to bring in design thinking to unpack mechanisms and contextual factors, rather than stopping at effect sizes.

## False promises of simulation

Yoshikawa et al. systematic review tackles a critical area of clinical education, infection control, by comparing simulation-based approaches with traditional teaching. As one reads through, a familiar pattern emerges knowledge improvement is the easy part; practice change is where the real challenge lies. While both traditional and simulation-based methods improved knowledge, simulation showed stronger effects for skills, confidence, decision-making, empathy, and self-reflection. However, the impact of these interventions was closely tied to the learner's level, scenario design, and the instructor's abilities, with instructors often ‘blamed' for variability rather than the modality itself. A timely reminder that modality alone does not change behavior.

## Mentorship beyond goodwill

Abdelmannan et al. conduct a scoping review endorsing the mentorship in graduate medical education in glowing terms but depict the messier realities eloquently. Academic health systems yearn for holistic mentorship practices, hoping they will influence not just individuals but the wider professional culture. Yet the review uncovers a patchwork of diverse mentorship programs relying heavily on individual goodwill and evaluated through narrow, research-focused outputs. The more abstract outcomes of identity formation, professional growth, and belonging remain largely intangible and theoretically underdeveloped. Theoretically grounded framework drawing on psychological and sociological traditions, offering practical guidance for conceptualizing responsive mentorship programs.

## Coaching as the next magic bullet

Bridging the persistent training–practice chasm has become a recurring theme in HPE, and academic coaching is now being positioned as the next magic bullet. King et al. offer a thoughtful perspective that reframes coaching not as the familiar ‘in-the-moment' sports model, but as ‘coaching over time' a longitudinal, self-regulation-oriented practice that supports learners as they navigate complexity. It's an enticing proposition, but one that demands deep sociocultural grounding and careful integration. And while the model is appealing on paper, its success relies on a rare breed of educators; reflective, altruistic, and detail-oriented the ‘Good Samaritans' of academic health systems. This paper highlights both the promise and the reality of coaching but our systems are not yet equipped to sustain it.

## Conclusion

Taken together, these eleven articles form a rich, textured mosaic of postgraduate and graduate HPE. Each piece illuminates a different corner of this landscape: curricular imagination, clinical learning spaces, digital infrastructures, instructional design, simulation practices, intergenerational realities, mentorship, and coaching. The picture that emerges is inspiring yet sobering—creativity abounds, but the field remains fragmented and theoretically thin. Across these contributions, a few threads emerge. First, PGME cannot simply inherit undergraduate structures and expect them to work. It requires its own deliberate, contextually grounded design. Second, faculty development, thoughtful pedagogy, and supportive infrastructures are not add-ons but essentials. Lastly, grand solutions like mentorship or coaching will falter without the slow, detailed, human work of building cultures that sustain them. PGME has far too long sat in the shadows. The honesty of these papers lies in the recognition that they don't offer silver bullets, but do offer silver linings of clarity, structure, and possibility. This Research Topic brings it, unapologetically, into the light.

A note on terminology: This Research Topic uses both postgraduate and graduate education to reflect international variations, with postgraduate typically referring to education following a first professional degree in European, Asian, and African contexts, while North American institutions use graduate education. We use these terms interchangeably to embrace the Research Topic's global scope.

